# The hypusine pathway in *Ixode*s *ricinus*: molecular cloning and validation of deoxyhypusine synthase as a novel target for drug discovery to treat and prevent vector borne diseases

**DOI:** 10.3389/fcimb.2025.1638906

**Published:** 2025-08-27

**Authors:** Annette Kaiser, Enzo Agostinelli, Dimitrios Tsikas

**Affiliations:** ^1^ Medical Research Centre, Universität Duisburg-Essen, Essen, Germany; ^2^ Department of Sensory Organs , Rheinische Hochschule Köln, Köln, Germany; ^3^ Department of Sensory Organs, Faculty Medicine and Dentistry, Sapienza University of Rome, Rome, Italy; ^4^ Core Unit Proteomics, Institute of Toxicology, Hannover Medical School, Hanover, Germany

**Keywords:** tick-borne diseases, *Ixodes ricinus*, hypusine, acaricides, deoxyhypusine synthase, eIF5A

## Abstract

Ticks are a group of arthropod vectors transmitting a variety of human pathogens, like *Borrelia* and the tick-borne Encephalitis virus. In Europe, *Ixodes* is the most important tick due to its wide distribution. Since the 20th century, *Ixodes* has significantly spread due to changes in biodiversity. Thus, there is an urgent need to decrease tick ubiquity in the environment to control tick-borne diseases.

Deoxyhypusine Synthase (DHS) catalyzes the first step in the post translational modification (PTM) of the amino acid hypusine in eukaryotic initiation factor (eIF5A). Modified eIF5A plays a crucial role in cell proliferation of different parasites. Therefore, we cloned a putative DHS locus of 1098 bp from *Ixodes* by a reverse genetic approach from total RNA of salivary glands and expressed the protein in *E. coli*. *Ixodes* DHS encodes an ORF of 365 amino acids and is commonly spread in different *Ixodes* (98.36%) and *Rhipicephalus* species (99%), and fruit flies (70.92%). The expressed DHS protein has a molecular weight of 40.88 kDa and a determined pI of 5.12. In an activity assay the enzyme shows moderate activity. In the future, we intend to perform virtual docking experiments once a 3D structure of *Ixodes ricinus* has been resolved to evaluate DHS as a novel target and to discover potent inhibitors to define its role in infection.

## Introduction

Ticks belong to a group of arthropod vectors, blood sucking ectoparasites that are characterized by transmission of a variety of pathogens. Mainly two important zoonotic diseases (Kahl and Gray, 2023) i.e., Lyme disease and Encephalitis virus are transmitted by *Ixodes ricinus*, the castor bean tick, which meanwhile occurs all over Europe due to climate changes (Semenza and Suk, 2018) and other factors. During blood feeding, ticks use the hypostome of their mouthpart to transmit saliva with inflammatory compounds and microorganisms. The saliva contains a plethora of components, i.e., cement proteins, lipocalins, salivary proteins (SALP proteins) to circumvent the immune defense of the host (Neelakanta and Sultana, 2022). *Ixodes ricinus* has four life cycle stages, i.e., egg, larvae, nymph and adult with three feeding stages on the vertebrate host (Kahl and Gray, 2023).

Ticks are the most important vectors in transmitting vector-borne diseases (Boulanger et al., 2019) They are responsible for transmission of bacteria, i.e., *Borrelia* and *Anaplasma*, and viruses of the group of Flaviviridae causing tick-borne encephalitis and parasites such as *Babesia* and *Theileria* (Boulanger et al., 2019). Mostly important with respect to human health, are zoonoses like Lyme disease caused by Borrelia and viral induced tick-borne encephalitis. In these cases, the human host is accidentally infected. Both infections cause an enormous economic burden with approximately 30 billion dollars per annuum in the US (Mac et al., 2019). Therefore, tick control by in depth studies of infection biology should foster the identification of novel targets and drugs.

In recent years we have intensively studied the biosynthesis of the unique PTM hypusine in eIF5A in the life cycle of different *Plasmodium* species (Kaiser et al., 2006). Deoxyhypusine synthase, which commits the first rate-limiting step of this pathway, transfers an aminobutyl moiety in a NAD^+^ dependent reaction from the triamine spermidine to the ϵ-amino group of a specific lysine residue in eIF5A (Umland et al., 2004). The *dhs* gene was identified in *Plasmodium falciparum* and *P. vivax* (Njuguna et al., 2006; Kaiser et al., 2007) respectively and shown to be involved in cell proliferation in different developmental stages of the parasite. Down-regulation of both genes could be demonstrated by *in vitro* knock downs (Schwentke et al., 2012) but knockouts of both genes suggested their vital function in murine malaria blood stage infection (Kersting et al., 2016).

Deoxyhypusine hydroxylase (DOHH) completes hypusine formation by introducing a hydroxyl group into the aminobutyl residue of deoxyhypusinated eIF5A, thus activating the precursor protein (Abbruzzese et al., 1988). The X-ray structure of human DOHH has been resolved (Han et al., 2015) and the protein shown to be a diiron enzyme with a peroxo-diiron III intermediate after reaction with O_2_. Most notably, the peroxo-diiron III intermediate has several longevity magnitudes compared to other peroxo intermediates (Han et al., 2015). In contrast, although DOHH was identified, cloned and expressed from *P. vivax* (Atemnkeng et al., 2013) its function in the *Plasmodium* life cycle remains unknown. Attempts to crystallize this protein are currently under way.

The important biological functions of hypusinated eIF5A are mainly attributed to its involvement of facilitating the translation of mRNAs with polyproline motifs (Gutierrez et al., 2013) and to nuclear cytoplasmatic shuttling (Rosorius et al., 1999; Templin et al., 2011). These findings, i.e., translation of polyproline specific m-RNAs, explain the participation of eIF5A and its cell type specific expression in a variety of human diseases (Guo et al., 2023; Kaiser, 2023). Moreover, the role of eIF5A in inflammation in pancreatic islets can be explained by shuttling cytokine inducing iNos2 mRNA to the cytosol for translation (Templin et al., 2011).

However, recent investigations showed that free hypusine deriving from proteolytic degradation of eIF5A, has biological activity (Tamborlin et al., 2023). In a C6 rat glioma cell line, hypusine reduced cell proliferation and decreased eIF5A transcript levels and global protein biosynthesis (Tamborlin et al., 2023).

Hitherto, there has only been one study performed on hypusine in Arthropods (Patel et al., 2009). This investigation focused on a homolog of DOHH called nero in *Drosophila melanogaster*. Knockdown experiments of either *nero* or *eIF5A* showed that both genes are required for cell cycle regulation, autophagy and protein synthesis (Patel et al., 2009). Moreover, *nero* mutations affected the development of sensory organs and the bristle size.

Ticks cause an elevating economic impact due to their transmission of pathogens and their increasing resistance against commercial acaricides (Obaid et al., 2022). The main reasons for the developing resistance are amino acid substitutions in the acaricide target, metabolic detoxification, and reduced uptake in the tick body. One of the main options for the future will be to understand the underlying mechanisms causing resistance against synthetic acaricides. In this context, the discovery of novel drug targets might be an important strategy providing perspectives for resistance prevention. Since DHS is essential for proliferation in many parasites, we cloned the *dhs* gene from the European tick *Ixodes ricinus* to validate it as a possible, novel target against different acaricides.

## Materials and methods

### Isolation of tick RNA

For the isolation of tick RNA a modified protocol from Cafiso et al (Urbanovâ et al., 2022). was applied.

A total of 20 adult female *Ixodes ricinus ticks* from each group took their blood meal on BALB/c mice (Urbanovâ et al., 2022) for either 12 or 24 hours. The inbred, pathogen-free BALB/c (The Jackson Laboratory, Bar Harbor, ME, USA) were purchased from Anlab (Prague, Czech Republic). In parallel, 21 nymphs were treated in the same way. Samples were stored alive in 50 ml conical tubes to avoid nucleic acids degradation. They were processed the same day using TRIzol™LS reagent (Thermo Fisher, Darmstadt, Germany) under a chemical fume-hood. Single tick individuals were washed in 1X PBS and dried on paper towels, identified under a stereomicroscope (Cafiso et al., 2021), and immediately processed. Thereafter, salivary gland material was prepared. RNA was isolated from salivary gland material with TRIzol™LS according to a protocol from Thermo Fisher Scientific, Darmstadt, Germany.

Salivary gland material (10–50 mg) was extracted with TRIzol™LS reagent (750 μL) and homogenized several times. An incubation period of 5 min.followed at room temperature (RT) to permit dissociation of the nucleoprotein complex. Chloroform (0.2 mL) was added per 0.75 mLof TRIzol™LS reagent. An incubation period of 2–3 min. followed. Thereafter, a centrifugation step followed at 12.000 rpm for 15min. at 4°C. After separation, the aequous, white phase containing the RNA was pipetted out and used for RT PCR reaction.

### Quantification of nucleic acids

Quantification of RNA or DNA was performed in a Nanodrop/UV/Vis spectrophotometer (Thermo Fisher. Scientific, Darmstadt, Germany), at 260 nm.

#### RT PCR and cDNA synthesis

RT-PCR and cDNA synthesis were performed according to a protocol from the Access Kit (Promega, Madison, Wisconsin, USA). The reaction in a volume of 50 μL contained: 23μL Nuclease free water, 10μl AMV/Tfl 5X Reaction Buffer, 1μl dNTP Mix (10mM each dNTP), 25mM MgSO4, 1μl AMV Reverse Transcriptase (5u/μl), 1μl Tfl DNA Polymerase (5u/μl), 10 μl total RNA (2,0 μg). 50 pmol of each upstream and downstream primer were applied. The upstream primer consists of an EcoRI 5’ flank <ns/>-5’GAATTCGCATGAGTGCCGAAGGAG-3’ and the downstream primer <ns/>-5’-TCACTTCACGGCGT CGCCGGCG-3’ contains a NotI 3’ flank, respectively. The primers containing the *EcoRI* and *Not I* restriction sites have been designed (Eurofins, Munich, Germany) for further subcloning into pet 28a (+) containing both enzymes in the cloning site. The reverse transcription was performed at 45°C for 45 minutes, followed by 1 cycle at 94°C for 2 minutes of AMV-RT inactivation and RNA/cDNA/primer denaturation. For second strand cDNA synthesis the following amplification steps were used: Denaturation was applied for 30s at 94°C. Annealing started at 60°C followed by extension at 68°C for 2 min for 40 cycles. A final extension step was performed at 68°C for 7 min. The reaction was cooled at 4°C. The obtained fragment of 1114 bp was sequenced by Eurofins, Munich, Germany.

#### Validation of signal intensities after RT-PCR analysis

Monitoring of signal intensities after RT-PCR analysis was performed by a GelStick Imager from Intas, Göttingen, Germany. To calculate the intensity of the signals, a tick reference gene glyceral-aldehyde-3-phosphate dehydrogenase *gapdh* was applied (Kôci et al., 2013) using two primers, i.e., primer forward F:5’-AGTGAAGCGTGTCCATCG-3’and primer R: 5’-GGGTAAACCTTGT

TGTCTGC-3’ resulting in an amplificate of 140 bp in size (original size of *gapdh* is 1311 bp, X.002434302).

#### Subcloning and expression of the putative dhs locus in pET-28a in E. coli BL21(DE3) cells and subsequent purification by Nickel-chelate chromatography

The sequenced fragment of 1114 bp with 5’ and 3’ flanks containing the *Eco*RI and *Not*I sites respectively, was cloned after restriction with *EcoRI* and *NotI* into *Eco*RI and *Not*I digested pET-28a (+) vector, carrying a N-terminal His•Tag/thrombin/T7•Tag for affinity purification by Nickel-chelate chromatography. The obtained recombinant plasmid was resequenced (Eurofins, Munich, Germany and transformed into *E.coli* BL21(DE3) cells.

For expression, the *E.coli* BL21(DE3) strain harboring the expression plasmid was grown in LB-medium with kanamycin (15μg.L^-1^) and expression was induced with IPTG (1mM).

Samples of 1 mL were taken for protein expression kinetics and centrifuged at 13.000 rpm for 2 min. After cell lysis with 400 μL lysis buffer, (50 mM Tris/HCl, pH 8.0) cells were centrifuged, then resuspended in lysis buffer and sonification was performed at 4°C for 30 s using a Branson sonifier (tip1 at 50%). After centrifugation for 10 min at 16.000 rpm at 4°C, samples were diluted in Roti® Load Gel loading buffer (4-fold) (Carl Roth, Karlsruhe, Germany) to a 1-fold concentration and run on a 10% SDS polyacrylamide gel at 100 V.

Nickel chelate affinity chromatography was performed under native conditions according to the Quiagen protocol with some modifications. A 5 mL culture was used from *E.coli* strain BL21 (DE3) expressing putative DHS from *Ixodes ricinus*. The thawed cells were resuspended in 700 μL lysis buffer (50 mM NaH_2_PO_4_, 300 mM NaCl, 10 mM imidazole, pH 8.0) for purification under native conditions and 70 μL lysozyme solution (10 mg/ml), 3 units benzonase for every mL of the original cell culture volume (15 Units in total) were added. An incubation for 30 min on ice followed. Thereafter, centrifugation was performed for 30 min at 15.000 rpm and the cleared lysate was loaded onto a Nickel NTA-spin column pre-equilibrated in lysis buffer. Next, the sample was centrifuged for 5 min at 15.000 rpm. The column was washed twice with 600 μL washing buffer containing 50 mM NaH_2_PO_4_, 300 mM NaCl, 20 mM imidazole, pH 8.0. Centrifugation was performed twice at 15.000 rpm for 5 min. The DHS from *Ixodes ricinus* was eluted from the column with 300 μL elution buffer 50 mM NaH_2_PO_4_, 300 mM NaCl, 500 mM imidazole in 2 fractions. Before a DHS activity assay, the imidazole was removed by dialysis for 12 hours and rebuffering with glycine NaOH buffer was performed.

#### DHS activity assay from *Ixodes ricinus*

A typical DHS activity assay contained in a total volume of 100 μL: 28 μg purified DHS protein from *Ixodes ricinus*, 40 μg human eIF5A protein, 2.1 mM spermidine, 3 mM NAD, 68.8 µL glycine NaOH buffer (Njuguna et al., 2006; Kaiser et al., 2007). After incubation at 37°C for 1 h, the reaction was stopped by freezing the sample. Human eIF5A precursor protein was used since the eIF5A precursor protein has not been cloned yet. The read out of the assay is described under GC/MS conditions (Baskal et al., 2022).

#### GC/MS conditions

To detect deoxyhypusine in the samples GC/MS analysis was employed (Baskal et al., 2022). GC/MS analysis was performed by GC/MS mass spectrometry consisting of a single-stage quadrupole mass spectrometer model ISQ, a Trace 1210 series gas chromatograph and an AS1310 autosampler from ThermoFisher (Dreieich, Germany). Toluene extracts (1 μL) were injected into the split- less mode with a 10μL Hamilton needle of the auto-sampler which was cleaned automatically three times with toluene (5 μL) after each injection. The injector temperature was kept at 280°C. Helium was used as the carrier gas at a constant flow rate of 1.0 mL/min. For qualitative analysis, the following GC program was used: the oven temperature was held at 40°C for 0.5 min and ramped to 320°C at a rate of 15°C/min which was held at this temperature for 1 min. Interface and ion-source temperatures were set to 300°C and 250°C, respectively. Electron energy was 70 eV and electron current 50 μA. Methane was used as the buffer reactant gas for NICI at a constant flow rate of 2.4 mL/min. The electron multiplier voltage was routinely set to 1400 V. Negative-ion chemical ionization and scanning in the *m*/*z* range 100–1000 (1 s per cycle) were performed.

#### Derivatization of deoxyhypusine

Derivatization of deoxyhypusine was performed according to a protocol by Baskal et al., 2022 (Baskal et al., 2022). Deoxyhypusine was either enzymatically and chemically synthesized and a kind gift from Dr. Kohout, University of Prague, Czech Republique and checked by high resolution mass spectrometry (HRMS). Deoxyhypusine was derivatized with tetradeutero-methanol (CD3OD; declared isotopic purity, ≥ 99.8% at 2H) (Sigma Aldrich, Steinheim) for methyl esterification of the amino group and penta-fluoro-propionic anhydride (PFPA) for acetylation of the carboxylic group. Methyl esters of deoxyhypusine were prepared according to the following protocol. Aqueous solutions of deoxyhypusine (0–10 μL, 10 mM) were evaporated to dryness under a stream of nitrogen. Subsequently, the solid residues were reconstituted in 100-μL aliquots of 2 M HCl/CH3OH or 2 M HCl/CD3OD solutions and esterification was performed by heating the samples for 60 min at 80°C. After cooling to room temperature, the solvents of the samples containing d0Me-Hyp and d3Me-Hyp were evaporated to dryness under a stream of nitrogen and subsequently, 100-μL aliquots of the PFPA/EA were added. The reaction mixture was heated for 30 min at 65°C to prepare penta-fluoro-propionic (PFP) derivatives. After cooling to room temperature, solvents and reagents were evaporated. The solid residues were treated first with 200-μL aliquots of 400 mM borate buffer, pH 8.5, and immediately thereafter with 200-μL aliquots of toluene, followed by immediate vortex-mixing for 60 s and centrifugation (4000×*g*, 5 min, 18°C). This step eliminates potential acidic components from hydrolyzed and reacted PFPA such as penta-fluoropropionic acid and extracts the methyl ester pentafluoropropionyl (Me-PFP) derivatives into toluene. Aliquots (150 μL) of the upper organic phase were subjected to GC/MS analysis.

#### Preparation of inhibitors

For inhibitor experiments Frontline® for small dogs was applied which consists of Fipronil (67 mg/1.34 mL). From the spot on solution 1μL was diluted in DFMO. Amitraz was obtained in a commercially available solution of 9% in water. Advantix® consists of Imidacloprid (100 mg) and permethrin (500 mg) in a solution of 2.5 mL butylhydroxytuluol (E312), N-methylpyrrolidon, citric acid and Miglyol 812. A 1:1000 solution was applied for the inhibitor experiments.

## Results

### Monitoring transcript levels of the putative dhs gene from Ixodes ricinus in salivary gland tissues

**Figure 1:**
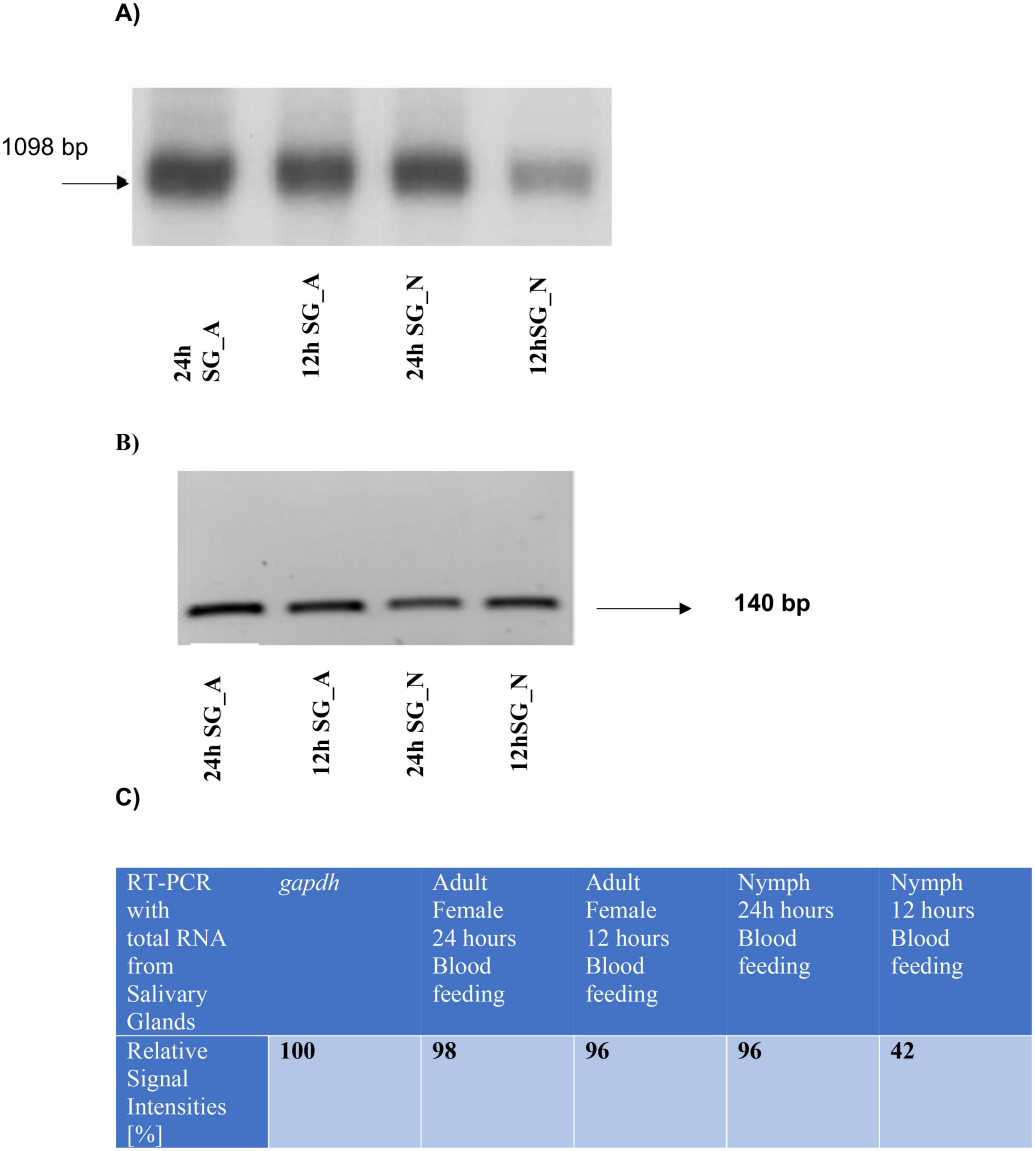
**(A)** Monitoring transcript levels by RT PCR with gene specific *dhs* primers obtained from total RNA extracted from salivary glands of nymphs or female, adult ticks after 12 or 24 h of blood feeding on mice. A transcript of 1098 bp was obtained independent of its stage-specific and time-dependent transcription. **(B)** Transcript levels of the reference control gene *gapdh* from the black leg tick *Ixodes scapularis* resulted in a band of 140 bp from total RNA extracted from salivary glands from nymphs or female adult ticks after 12 or 24h of blood feeding. **(C)** Densitometric quantification of signal intensities obtained from RT PCR reactions with *dhs* primers from total RNA extracted from salivary glands of nymphs or adult female ticks after different time points of blood feeding.

Based on recent data from a transcriptome profiling in salivary glands and midgut tissues from *Ixodes ricinus* (Kotsyfakis et al., 2015) (Access. No. GADI01002538), we performed RT-PCR analysis with gene specific primers from a putative *dhs* gene using salivary tissue from either nymphs or adults after blood feeding for 12 h or 24 hours on BALB/c mice (**Figure 1A**). The most intensive signal of the 1098 bp transcript was detected in adult and nymph tissue from salivary glands after 24 hours of blood feeding in mice. In contrast a significant lower *dhs* transcript level was observed in nymphs after 12 hours of blood feeding. To validate our results, glyceraldehyde 3-phosphate dehydrogenase (*gapdh*) from the black leg tick *Ixodes scapularis* (Kôci et al., 2013) was applied as a reference gene in RT-PCR reactions using total RNA from salivary glands from nymph and adult female ticks after 12 h and 24 h of blood feeding (**Figure 1B**). While the *gapdh* gene showed no difference in signal intensities (**Figures 1B, C**), transcript levels of the *dhs* gene from *Ixodes ricinus* varied as depicted in **Figure 1C** representing the results of a densitometric analysis of signal intensities. These results suggest that the putative *dhs gene* is transcribed in a stage- specific and time-dependent manner.

### Molecular cloning and characterization of the dhs gene from *Ixodes ricinus*

A bio-informatics screening of different nucleic acid and transcriptome databases (Baskal et al., 2022) led to the identification of an ORF of 365 amino acids encoded by 1098 bp from salivary gland tissue of adult and nymphs from *Ixodes ricinus.* Therefore, we used a reverse genetic approach to amplify the putative *dhs* gene with gene specific primers from total RNA isolated from salivary gland material obtained from female adults after 24 h of blood feeding.

**Figure 2:**
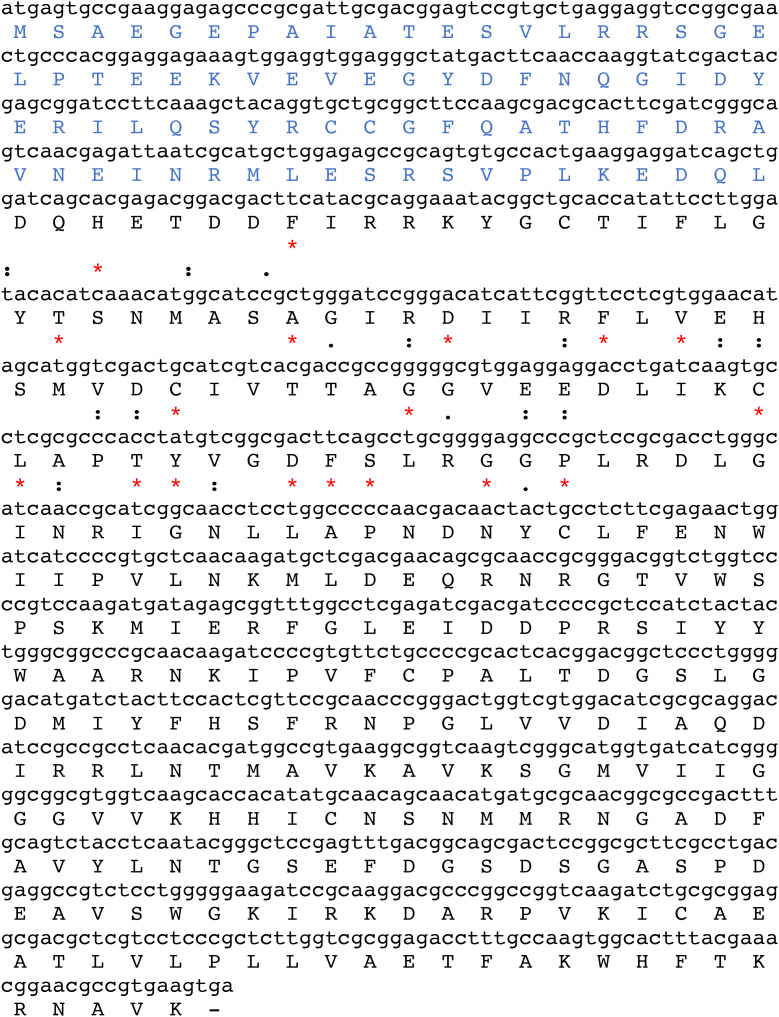
The ORF of 1098 bp encoded by the putative DHS nucleic acid sequence from *Ixodes ricinus* comprises 365 amino acids. A stretch of the nucleic acid sequence of the putative *dhs* gene (position 144-216) shows 56% identity to the chemokine binding protein Evasin P1128. Identities are marked as asterisks (*) while similarities are represented as colons (): and dots (.).

The sequence of the amplified fragment of 1098 bp generated after RT-PCR showed 100% sequence identity to the “putative dhs” locus found in the transcriptome databases (**Figure 2**). Surprisingly, a stretch of amino acid identity with approximately 57% homology was identified to Evasin P1128 and less homology to Evasin P1025 (47,8%) and Evasin P1026 (43,50%) respectively (**Figure 2**). Evasins are chemokine binding proteins which occur only in ticks and suppress chemokine-mediated inflammation (Mans et al., 2022). Chemokines are secretory proteins with different spacing between the N-terminal cysteine residues which interact through recognition sites with G-protein coupled receptors. Chemokine binding specificity of Evasins is controlled by a knottin scaffold protein (Lee et al., 2019). The striking homology in a stretch of sequence of the *dhs* gene to different Evasins may suggest a role in immunomodulation during blood feeding of the human host.

**Figure 3:**
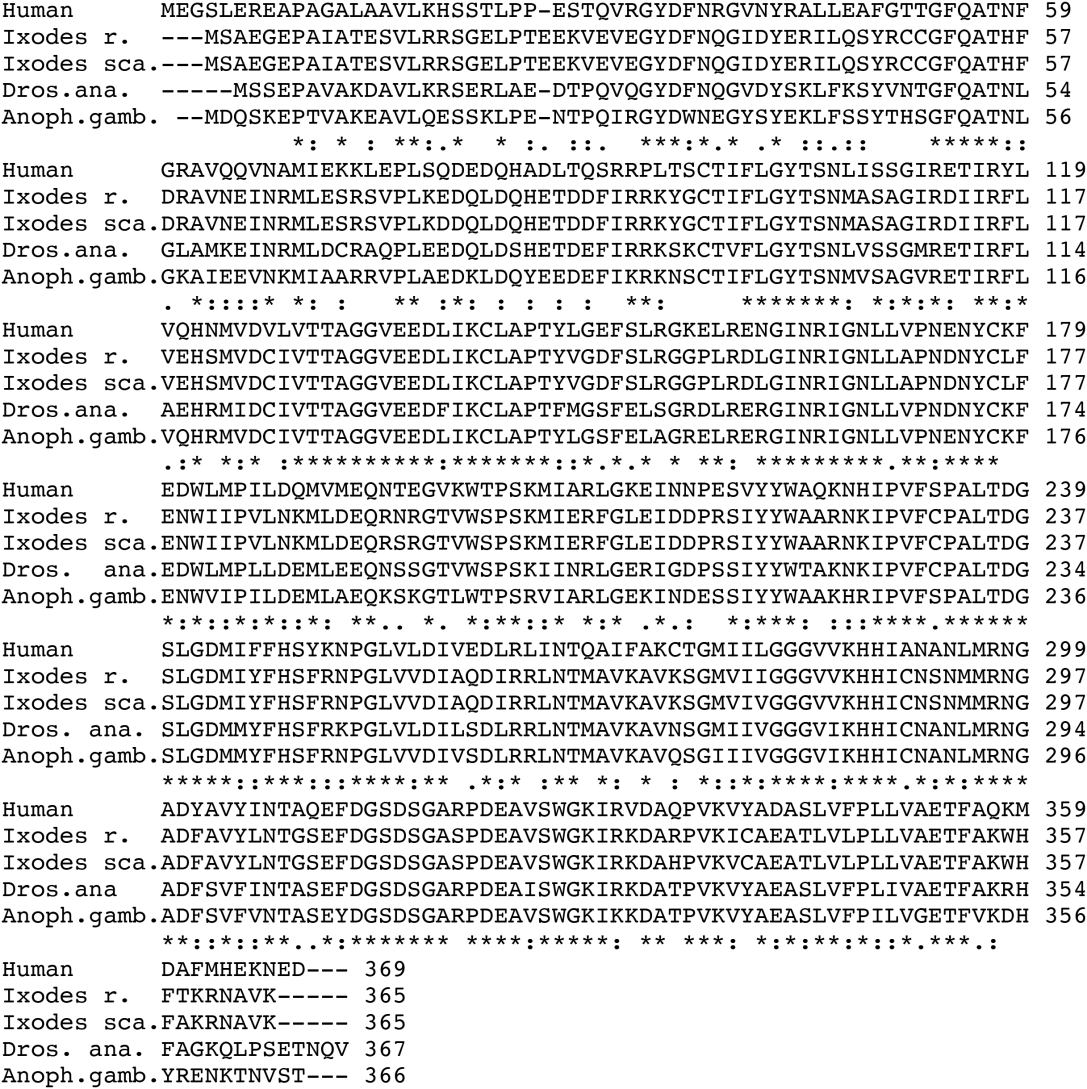
Multiple amino acid alignment from *Ixodes ricinus* DHS and three different vectors, i.e., *Ixodes scapularis, Drosophila ananaessa, Anopheles gambiae a*nd the human paralogue. Gaps (-) were introduced to obtain maximum alignment. Asterisks label amino acid identities, colons (): and dots (.) represent amino acid similarities (Madeira et al., 2024).

**Figure 4:**
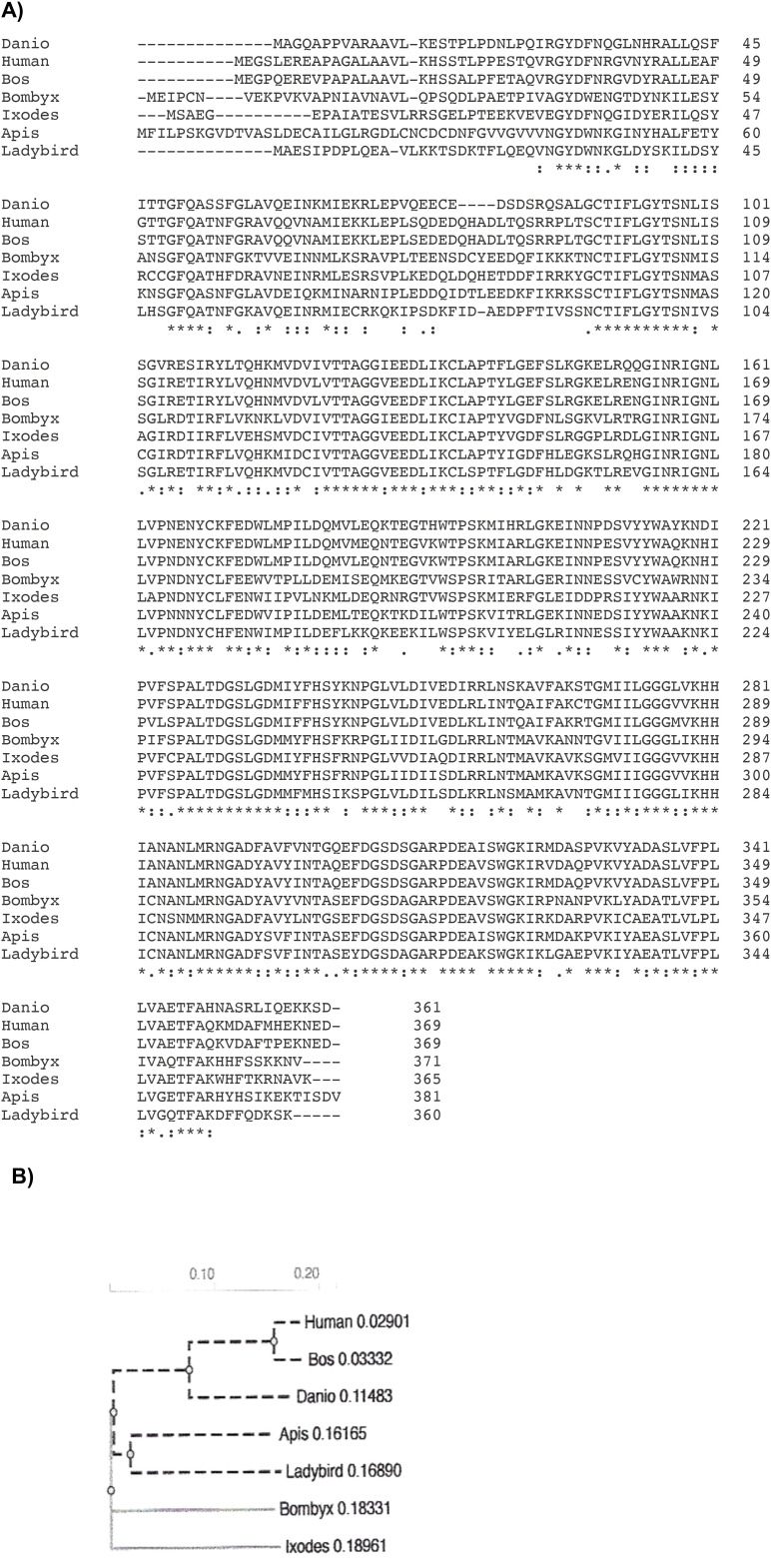
**(A)** A multiple amino acid alignment from *Ixodes ricinus* DHS to DHS from beneficial Arthropods like ladybird (*Cochinella septempunctata*), a bee (*Apis mellifera*), a silk moth *Bombyx mori* and the vertebrates zebrafish *Danio rerio*, *Bos taurus* and *Homo sapiens.* The alignment was applied to perform a phylogram. Asterisks label amino acid identities, colons (): and dots (.) represent amino acid similarities (Madeira et al., 2024). **(B)** A phylogram presenting evolutionary relationship of DHS from *Ixodes rizinus* in Arthropods and Vertebrates. Analysis was performed with ClustalW Omega (Madeira et al., 2024) to calculate the tree distance. The most common recent ancestors of *Ixodes Ricinus* are *Bombyx mori*, *Apis mellifera*, and the beatle *Cocccinella septempunctata*.

A multiple sequence alignment shows (**Figure 3**) that DHS is widely spread among different *Ixodes* species with 98% amino acid identity to the black leg tick *Ixodes scapularis*. DHS shares 68.7% homology to the fruitfly *Drosophila ananaessa* and 66% homology to *Anopheles gambiae*, the principal malaria vector in Africa (Takeen et al., 2024). 60% homology on the amino acid level was detected for the human DHS protein. To further strengthen our observation that DHS from *Ixodes ricinus* has highly conserved motifs in arthropods and vertebrates, we performed a phylogenetic analysis between different beneficial arthropods like ladybird (*Cochinella septempunctata*), a bee (*Apis mellifera*), a silk moth *Bombyx mori* and the vertebrates zebrafish *Danio rerio*, *Bos taurus* and *Homo sapiens* (**Figure 4A**).The results presented in the phylogram suggest a close relationship of DHS between representatives of the different arthropods and an early common ancestor (second node on the left side in **Figure 4B**) for the vertebrates, i.e, zebrafish, *Danio rerio* and the closely related lineages of *Homo sapiens* and *Bos taurus*.

### Expression, purification of DHS from *Ixodes ricinus* and functional analysis of the protein

**Figure 5:**
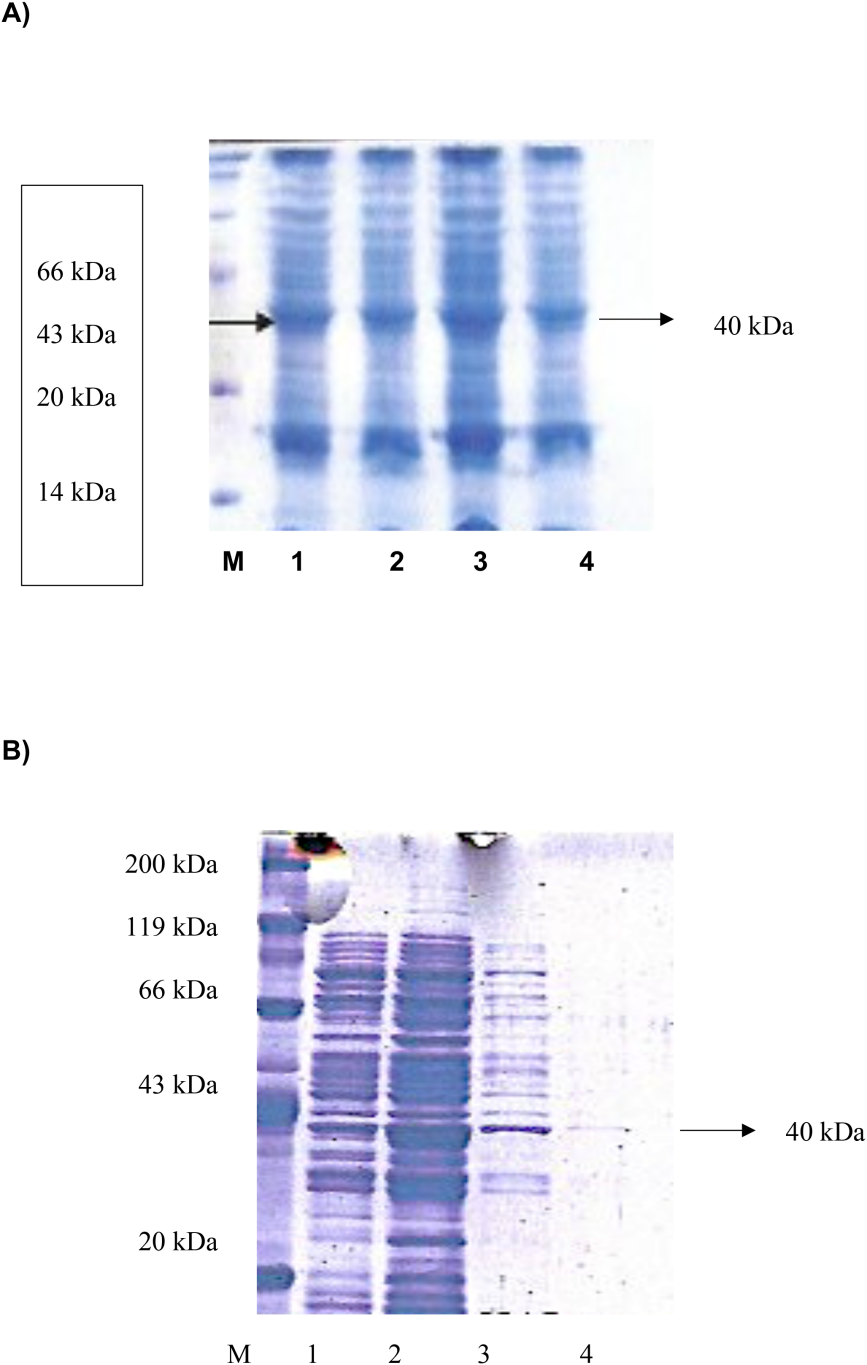
**(A)** Expression profiling of recombinant histidine-tagged DHS from *Ixodes ricinus* between 1 and 4 hours after IPTG induction. M, protein standard marker. **(B)** Expression and purification of recombinant histidine-tagged DHS from *Ixodes ricinus* by Nickel-chelate affinity chromatography under native conditions. M, Roti standard protein marker; 1, lysed crude cell extract; 2, Wash fraction; 3, Eluate fraction 1 containing recombinant DHS protein; 4, Eluate fraction 2.

Expression of the recombinant DHS protein was performed in pET28a(+) vector in *E. coli* BL21(DE3) cells harboring T7 RNA polymerase under the control of the T7 promotor. Expression profiling resulted in maximum expression levels after 3 hours of IPTG induction. (**Figure 5A**, lane 3). Next, purification of the DHS protein was performed by nickel chelate affinity chromatography since the N-terminus of the protein consists of a histidine tag and enables binding to the affinity column. After expression and purification under native conditions a protein of 40 kDa (predicted size) was detected in both eluate fractions (**Figure 5B**, lanes 3 and 4).

As proof of principle DHS activity was determined with the purified protein by nickel chelate affinity chromatography and analyzed by GC/MS analysis (**Table 1**). The enzyme assay contained purified human eIF5A protein (40 μg), 2.1mM spermidine as substrate, purified DHS protein (28 μg), 3.0 mM NAD cofactor in a total volume of 100 μL. Most notably, purified eIF5A precursor protein from human was accepted as a substrate from *Ixodes ricinus* DHS since the *eIF5A* gene from the tick has not been cloned yet. However, previous experiments have shown that eIF5A has a broad substrate specificity and its interaction with different proteins has been shown (Sievert et al., 2012). Moreover, recent data showed that there is a measurable release of enzyme intermediate bound NADH to catalyze the reaction without the precursor protein eIF5A (Park et al., 2017). DHS specific activity from the tick (185 pmol/mg protein) was lower than human DHS (200 pmol/mg protein) and DHS from *P.vivax* (237 pmol/mg protein) (**Table 1**). No DHS activity was detected in control experiments without either the precursor protein, the DHS protein, or the substrate spermidine.

**Table 1:** A comparison of specific DHS activities between different organisms.

DHS activity assay:		
DHS	28 μg	
eIF5A	40 μg	
Spermidine	2.1 mM	
0.5 M NAD	3.0 mM	
Glycine NAOH buffer	68.8 μL	
Specific DHS activity [pktal/mg protein]	Organism	EIF5A
237	*P.vivax*	*P.vivax*
200	*Homo sapiens*	*Human*
185	*Ixodes ricinus*	*Human*

Next, we analyzed the inhibitory effect of different acaricides on DHS activity to detect possible off target effects of the compounds. Frontline® consists of Fipronil, a phenylpyrazole which disrupts GABA-gated chloride channels (Simon-Delso et al., 2015) inhibiting the chloride influx leading to hyperexcitation of the ectoparasite’s nervous system and consequently to death. Advantix® is a drug combination of Imidacloprid (Taillebois et al., 2018) and Permethrin (Burtis et al., 2021). Imidacloprid is a neonicotinide that blocks acetylcholine receptors leading to damage of the insect’s nervous system and finally to death. Permethrin interferes with sodium channels to disrupt the function of neurons. DHS activity was inhibited to 48% by the drug combination of Imidacloprid and Permethrin in Advantix® while Fipronil reduced DHS activity to 44% respectively (**Table 2**), suggesting an interaction with a binding site for both drugs in the DHS protein. Lower DHS inhibition of 35% in comparison to the untreated control was determined for Amitraz, a foramidine blocking octopamine receptors in the tick (**Table 2**).

**Table 2:** Inhibition of DHS activity by various acaricides.

Inhibition of DHS activity [%]	Acaricide	Inhibitor concentration [μM]
44% ± 0.5%	Fipronil	67
48% ± 0.9%	Imidacloprid+permethrin	40 + 200
35% ± 0.4%	Amitraz	0.88
0%	————	—–

## Discussion

In an approach to study the function of DHS in a human, disabling ectoparasite, we identified the *dhs* gene from *Ixodes ricinus*, the most common tick in Europe. The protein has a molecular weight of 40.88 kDa and a pI of 5.62. Surprisingly, DHS from *Ixodes ricinus* has highly conserved amino acid regions compared to other insect vectors like the fruitfly *Drosophila ananaessa*, *Anopheles gambiae*, the principle vector in Africa and to human DHS. A phylogram (**Figure 4B**) representing this evolutionary conserved relationship to different beneficial arthropods and three different vertebrates strengthens this observation. The significant homology of 60% to the human orthologue means an enormous challenge in drug discovery to find selective inhibitors most probably in the unconserved regions of the active site of the protein. Moreover, intensive biochemical studies on the *Ixodes* DHS protein and crystallization experiments in combination with virtual docking experiments will provide necessary information for lead optimization. A first step in the biochemical direction will be the determination of dose response curves of the investigated inhibitors and determination of K_i_ values to clarify the “off target effects”.

The significant conservation of *Ixodes ricinus* DHS may suggest an essential, biological function in the life cycle of the tick. However, this hypothesis fosters a *dhs* gene edited tick (Nuss et al., 2021). Techniques to construct stable genetic germlines by CRISPR/Cas9 are in development and can only be achieved by microinjection into newly, deposited tick eggs (Sharma et al., 2022) or gravid female ticks. The first genetic experiments with CRISPR/Cas9 technologies have shown their feasibility (Sharma et al., 2022) but remain challenging. These innovative tools will be essential to understand host pathogen transmission by ticks and the biology of host pathogen interactions.

Activity assays showed that the expressed DHS protein from *Ixodes* is fully active but show minor activity compared to their homologues from human and *P.vivax*. Interestingly, DHS from *Ixodes* accepts the human eIF5A precursor protein as a substrate for modification due to a broad substrate specificity with different interacting proteins. This is caused by its high conservation throughout the species.

The most interesting result is the inhibitory and “off target” effect of different acaricides, i.e., Fipronil, Imidacloprid, Permethrin and Amitraz on DHS activity since they normally target structures in neurons, i.e., GABA-gated chloride channels, acetylcholine receptors, sodium channels and octopamine receptors, respectively. However, SPD, the substrate of DHS has been shown to promote hypusine formation in the *Drosophila* brain thus preventing age-mediated brain decay caused by a decline in respiratory chain function (Liang et al., 2021). Moreover, it was shown that SPD induces autophagy in a *Drosophila* age-model affecting the autophagy regulator Atg7 and mitophagy inducers Parkin and neuron specific Pink1 (Schroeder et al., 2021). Hitherto, we cannot exclude due to the absence of structural data that the acaricides, i.e., Fipronil, Imidacloprid and permethrin can bind to an allosteric site of the tick DHS since structural data of the DHS protein are missing.

**Figure 6:**
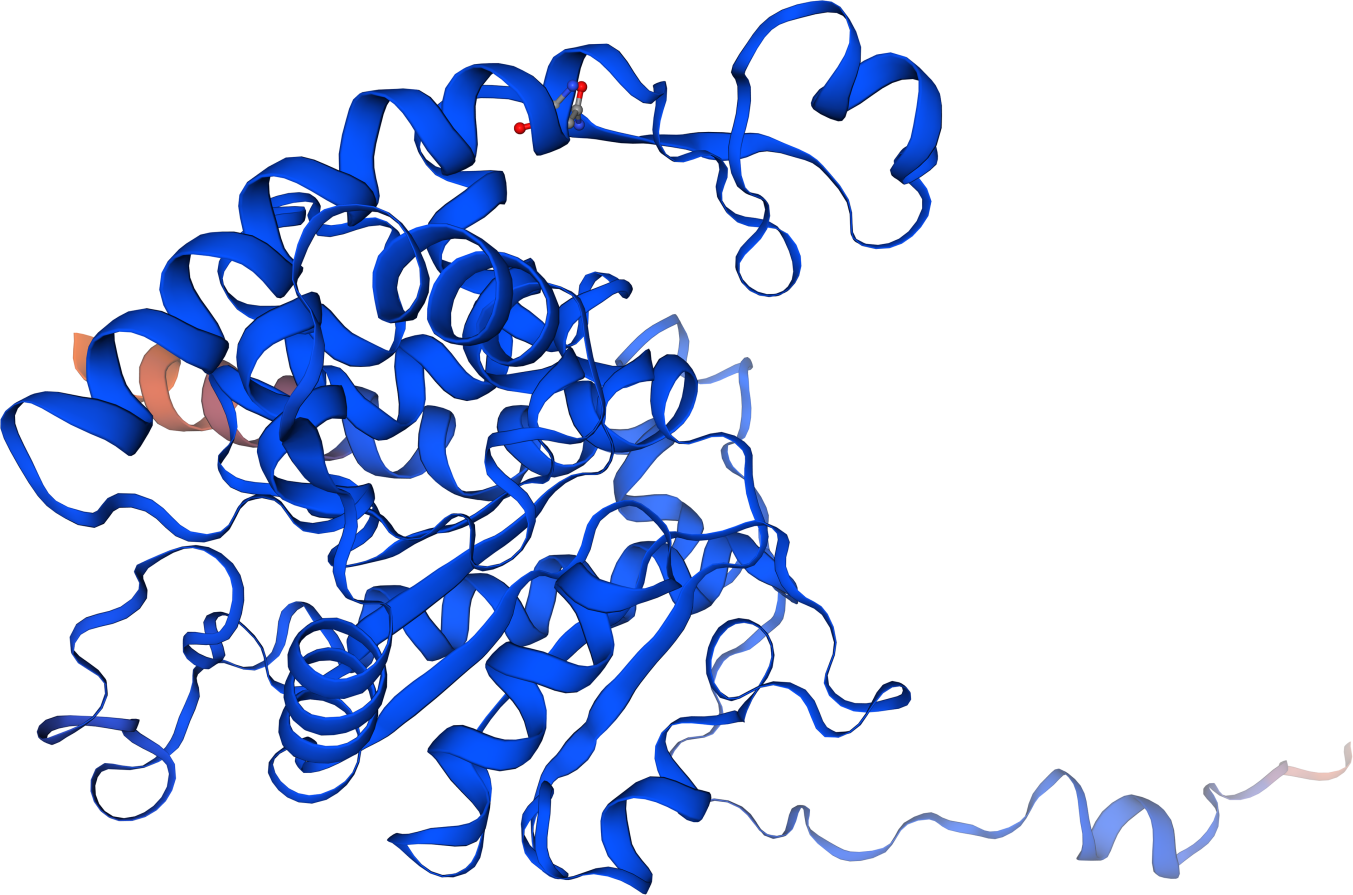
The Alphafold model structure of a putative DHS monomer from *Rhipicephalus pulchellus* based on 100% amino acid identity to DHS from *Ixodes rizinus* with a predictability score of 98.1%.

Interestingly, a screen of the Alphafold Database (Abramson et al., 2024) revealed a virtual model structure with 100% amino acid identity of the monomer of a putative DHS protein from *Rhipicephalus pulchellus*, the yellow backed tick, with an average model confidence of 95.81, suggesting very high confidence (**Figure 6**). Moreover, an average model confidence of 58 was obtained in a Swiss Model for DHS from crystallized *Trichomonas vaginalis* (Wątor et al., 2024). In this context, virtual docking experiments will support the identification of lead structures.

Monitoring of transcript levels (**Figure 1**) revealed that the *dhs* gene is differentially transcribed in the salivary glands depending on the developmental stage and time- specific blood feeding. The most intense signals for *dhs* transcripts were obtained after 24h of blood feeding for nymphs and adults. Tick feeding is a dynamic process with a pleiad of proteins, the sialome, located in salivary glands being involved. Blood feeding may last days for *Ixodidae* to modulate the host immune response, i.e., cytolytic, hematolytic and inflammatory processes. Recent proteomic investigations showed a shift of proteins and biological processes during the blood feeding process (Fernández-Ruiz and Estrada-Peña, 2022). Upregulation at the *dhs* transcript level might be a first step in this direction since hypusination of eIF5A is essential to maintain protein translation and thus might be essential for completion of the tick’s lifecycle.

The striking homology of *dhs* to Evasin P1128, a chemokine binding protein might pinpoint a further function in immunomodulation of the host response. This hypothesis is further strengthened by the fact that regulatory key enzymes of the polyamine pathway are involved in differentiation of CD4^+^ T cells into functional fates (Puleston et al., 2021). Thus, mice deficient in hypusine develop severe inflammatory diseases (Puleston et al., 2021).

It would be of further interest to investigate *dhs* transcription in the midgut since it has a pivotal role not only in blood meal digestion but also in pathogen acquisition and transmission (Lu et al., 2023).

## Data Availability

The datasets presented in this study can be found in online repositories. The names of the repository/repositories and accession number(s) can be found below: GADI01002538.1 putative locus given in the text.
